# Magnetic Properties of Strontium Hexaferrite Nanostructures Measured with Magnetic Force Microscopy

**DOI:** 10.1038/srep25985

**Published:** 2016-05-13

**Authors:** Qiang Li, Jie Song, Matilde Saura-Múzquiz, Flemming Besenbacher, Mogens Christensen, Mingdong Dong

**Affiliations:** 1 Interdisciplinary Nanoscience Center (iNANO), Aarhus University, Aarhus C, DK-8000, Denmark; 2 Key Laboratory of Colloid and Interface Chemistry, Ministry of Education, Shandong University, Jinan, 250100, P.R. China; 3 Center for Materials Crystallography, Department of Chemistry, Aarhus University, Aarhus C, DK-8000, Denmark

## Abstract

Magnetic property is one of the important properties of nanomaterials. Direct investigation of the magnetic property on the nanoscale is however challenging. Herein we present a quantitative measurement of the magnetic properties including the magnitude and the orientation of the magnetic moment of strontium hexaferrite (SrFe_12_O_19_) nanostructures using magnetic force microscopy (MFM) with nanoscale spatial resolution. The measured magnetic moments of the as-synthesized individual SrFe_12_O_19_ nanoplatelets are on the order of ~10^−16^ *emu*. The MFM measurements further confirm that the magnetic moment of SrFe_12_O_19_ nanoplatelets increases with increasing thickness of the nanoplatelet. In addition, the magnetization directions of nanoplatelets can be identified by the contrast of MFM frequency shift. Moreover, MFM frequency imaging clearly reveals the tiny magnetic structures of a compacted SrFe_12_O_19_ pellet. This work demonstrates the mesoscopic investigation of the intrinsic magnetic properties of materials has a potential in development of new magnetic nanomaterials in electrical and medical applications.

Nanoscale magnetic materials are attracting tremendous research interests due to their unusual properties compared to the bulk materials and their applications in many areas of science and technology^1^,^2^,^3^,^4^,^5^,^6^,^7^. M-type strontium hexaferrite (SrFe_12_O_19_) is an important hard magnetic material with a ferrimagnetic structure. Owing to its unique magnetic properties, it is very suitable for the use in data storage and electronic devices^8^. Bulk SrFe_12_O_19_ is traditionally used in the fabrication of permanent magnets and in the design of microwave devices operating at high frequencies because of its large axial magnetic anisotropy, high intrinsic coercivity and high permeability. In recent years, due to the new fundamental and emerging applications in electronics^9^, the research interest in SrFe_12_O_19_ has been renewed. SrFe_12_O_19_ nanomaterials can be used in the design of electronic components for automobile and wireless communications^8^,^10^. However, all of these innovative applications based on SrFe_12_O_19_ need nanoscale understanding and controlling of the magnetic properties such as the magnitude and orientation of the magnetic moment. It is also well-known that the magnetic properties of SrFe_12_O_19_ are strongly dependent on its nanostructure size, shape, orientation, and domain configurations^9^,^11^,^12^. Hence, direct investigation and characterization of SrFe_12_O_19_ nanostructures with high magnetic sensitivity and nanoscale spatial resolution is highly desirable to understand the origin of the magnetism of SrFe_12_O_19_ nanostructures.

Although sensitive techniques such as superconducting quantum interface device (SQUID) and vibrational sample magnetometer (VSM) have been developed for macroscopic measurements of the magnetic properties of magnetic materials, little has been done on the mesoscopic characterization of the magnetic properties of magnetic nanostructures. Thus so far, the direct measurement of magnetic nanostructures is only possible by using microscopy techniques. Magnetic force microscopy (MFM) is such a microscopy tool to detect and localize nanoscale magnetic domains utilizing the magnetic interactions between the magnetized probe and the sample^13^. Recent studies have demonstrated the abilities of the MFM to characterize magnetic nanoparticles with high magnetic sensitivity and spatial resolution similar to atomic force microscopy (AFM)^14^,^15^,^16^,^17^,^18^,^19^,^20^. Therefore, MFM is ideal to characterize the magnetic nanostructures of SrFe_12_O_19_.

In the present study, we demonstrate the quantitative imaging of the magnetic nanostructures of SrFe_12_O_19_ by employing MFM. Through addressing the crystal structure of SrFe_12_O_19_, the theory as well as the experimental practice of the MFM technique, we obtained a better understanding of the magnetism of the nanostructures of SrFe_12_O_19_. The experiments reveal that the magnetic moment of SrFe_12_O_19_ nanoplatelets depends on their thickness. In addition, the magnetization directions of SrFe_12_O_19_ nanoplatelets in the aggregates can be clearly identified by MFM frequency imaging. Furthermore, MFM frequency imaging clearly reveals the magnetic domains in a compacted SrFe_12_O_19_ pellet.

## Results

### Theory of MFM

MFM is a specialized operation mode of AFM that utilizes the relatively weak but long-range magnetic interactions between the magnetized probe and the sample while minimizing the influence of sample topography^13^. MFM measurements are taken in a dual-pass tapping/lift mode, meaning each line in the MFM image is the compilation of a tapping-mode scan and a lift-mode scan. In the first pass, the topography information was acquired in tapping mode. The tip is then lifted and the topography profile record from the tapping-mode scan is used to maintain a constant height (so-called lift height) between the tip and local surface topography. In this lifted position, the influence of magnetic force *F*(*z*) can be measured by directly tracking the shifts in resonant frequency of the tip, is given by


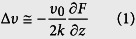


where Δ*υ* is the frequency shift; *υ*_0_ and *k* are the resonant frequency and the spring constant of the MFM cantilever, respectively. *∂F/∂z* is the force gradient.

In general, the magnetic force acting on the tip can be calculated through integrating the tip-sample force density over the tip volume or rather its magnetized part. In order to make the calculations feasible, simplified models for the tip magnetic structure are often used. The simplest way to model a tip is to assume the effective dipole moment of the tip is located in the center of a sphere approximating the tip apex. Thus the interaction between a spherical magnetic particle and a magnetic tip can be considered in a dipole-dipole model, given as^17^,^21^:


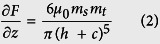


where *μ*_0_ is the vacuum permeability; *m*_s_ and *m*_t_ are the magnetic moments of the magnetic sample and the MFM tip, respectively; *h* is the lift height; *c* is a constant related to distance of the magnetic dipoles within the magnetic particle and MFM tip. By combining Equations (1) and (2), we find


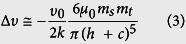


Therefore, the magnetic moment of the magnetic sample *m*_*s*_ can be obtained by measuring the MFM frequency shift Δ*υ*.

### Crystalline Structure and Macroscopic Magnetic Property of Strontium Hexaferrite

**Figure 1:**
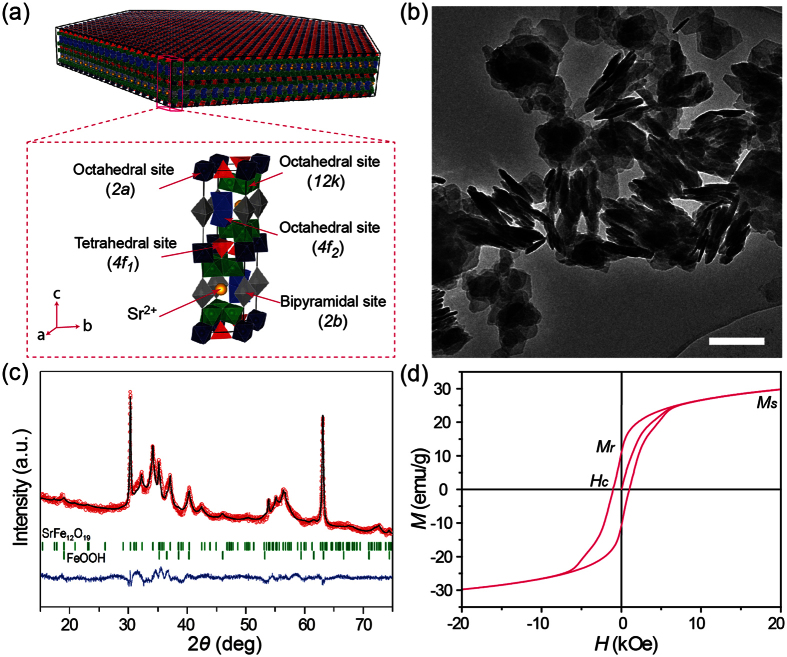
(**a**) Schematic hexagonal nanoplatelet and crystal structure of the M-type SrFe_12_O_19_. The polyhedra with different color depict the different Fe^3+^ sites with their surrounding O^2−^ ions. The yellow spheres depict the Sr^2+^ ions. (**b**) Bright-field TEM image of the as-synthesized SrFe_12_O_19_ samples, confirming the hexagonal nanoplatelet morphology. The scale bar is 200 nm. (**c**) The Rietveld refinement of powder XRD pattern of the as-synthesized SrFe_12_O_19_ samples. The red line represents the experimental data, and the black line is the calculated pattern; the green vertical bars are the expected Bragg reflection positions, and the difference between the experimental data and the calculated pattern is shown in blue at the bottom. (**d**) *M-H* hysteresis loop for the as-synthesized SrFe_12_O_19_ samples measured at 300 K.

Figure 1a presents the schematic crystal structure of the M-type SrFe_12_O_19_. The hexagonal structure can be considered to be made up of alternating spinel (S = Fe_6_O_8_^2+^) and hexagonal (R = SrFe_6_O_11_^2−^) layers. The O^2−^ ions are closed packed with the Sr^2+^ ion in the hexagonal layer and the Fe^3+^ ions are distributed in the octahedral (12 k, 2a and 4f_2_), trigonal bipyramidal (2b) and tetrahedral (4f_1_) sites. The magnetic moments of the Fe^3+^ ions are coupled to each other by super-exchange interactions through the O^2−^ ions. The Sr^2+^ ion is responsible for the large magnetic uniaxial anisotropy as it causes a perturbation of the crystal lattice^9^. In this study, the SrFe_12_O_19_ samples were synthesized by supercritical flow synthesis^22^. Figure 1b shows a typical bright-field transmission electron microscopy (TEM) image of the as-synthesized SrFe_12_O_19_ samples. Hexagonal nanoplatelets with a plate diameter of <100 nm can be clearly observed. It can also be observed that some of the nanoplatelets are superimposed over each other forming stacked nanoplatelets. This is most likely owing to the magnetic interactions between nanoplatelets as the crystallographic c-axis is the magnetic easy axis. The room temperature powder X-ray diffraction (XRD) pattern and Rietveld refinement of the as-synthesized SrFe_12_O_19_ samples is shown in Fig. 1c. The results obtained from refinements show the SrFe_12_O_19_ to be the main phase present (89 weight%), refined as the magnetoplumbite structure with space group of *P6*_*3*_*/mmc*. The refined lattice parameter values (a = b = 5.8887(2) Å and c = 23.101(4) Å) are in good agreement with the previous reports for SrFe_12_O_19_^23^,^24^. The refined crystallite sizes (of 30.2(4) nm along a- and b- axes and 2.66(3) nm along c axis) extracted from the diffraction data are comparable in magnitude to the nanoplatelet sizes observed in TEM. A secondary phase is also present in the sample. It constitutes 11 weight% and it was identified and refined as the defect-free FeOOH structure reported by Jensen *et al*.^25^ with space group of *P*-31c. The FeOOH phase is also found forming hexagonal nanoplatelets, of similar refined sizes (18(2) nm along a- and b- axes and 6.4(6) nm along c axis) to those of SrFe_12_O_19_. In order to measure the macroscopic magnetic properties of the as-synthesized SrFe_12_O_19_ samples, magnetization-field (*M-H*) hysteresis loop was performed by VSM at 300 K as shown in Fig. 1d. It is clear that the sample is a hard magnetic material at room temperature with the saturation magnetization (*M*_s_) of about 30 emu/g at *H* = 20 kOe. The remanence magnetization (*M*_r_) and the intrinsic coercivity (*H*_c_) extracted from the hysteresis loop are of 11 emu/g and 1 kOe, respectively.

### Probing the Magnetic Properties by MFM at the Nanoscale

**Figure 2:**
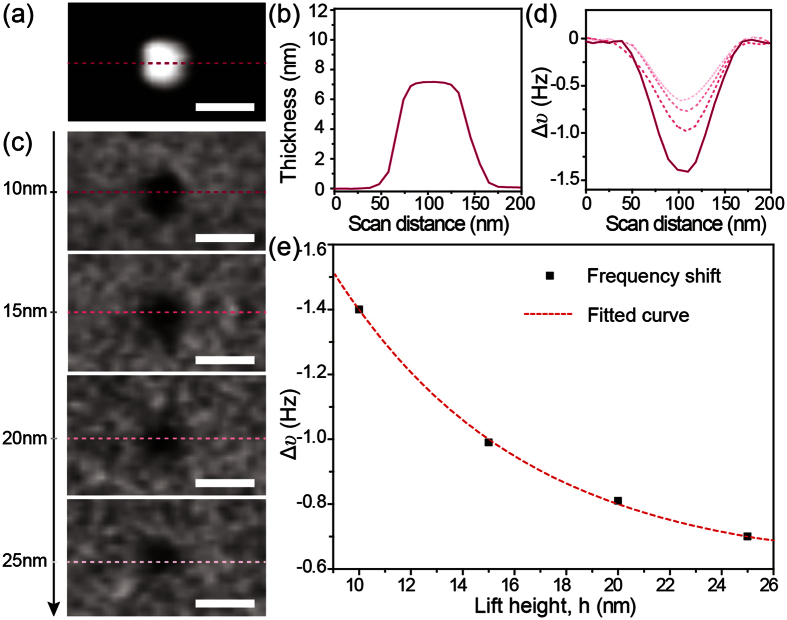
(**a**) AFM height image of a SrFe_12_O_19_ nanoplatelet and (**b**) height profile along the dashed line marked in (**a**). (**c**) MFM frequency images and (**d**) frequency shift profiles measured at different lift heights h = 10, 15, 20, 25 nm. (**e**) Obtained frequency shifts as a function of lift height. The black square symbols correspond to the maximum frequency shift obtained from the frequency images in (**c**). The dashed red line is the fit of the frequency shift value using Equation 3. All scale bars are 100 nm.

The VSM measurements confirmed the magnetic nature of the SrFe_12_O_19_ sample. However, the VSM method only allows the measurement of a macroscopic sample, *i.e.* integral properties of ensembles of SrFe_12_O_19_ nanoplatelets. MFM was employed for the direct characterization of the magnetic properties of individual SrFe_12_O_19_ nanoplatelets. In this study, frequency modulation is used to track the shifts in resonant frequency due to its high sensitivity to the magnetic force gradient (Figure S1). Figure 2 presents the results of MFM measurements of the magnetic properties of a SrFe_12_O_19_ nanoplatelet. The AFM height image (Fig. 2a) shows the nanoplatelet with a diameter of about 100 nm and a thickness of about 7.1 nm, as can be seen more clearly from the height profile through the center of the nanoplatelet (Fig. 2b). Magnetic force gradient images (shown as frequency images) of the same nanoplatelet recorded at different lift heights are shown in Fig. 2c (see also Figure S2). As can be seen, the frequency contrast of the nanoplatelet decreases as the lift height increases. This is clearly evident from the frequency shift profiles (Fig. 2d) taken along the dashed lines marked in the frequency images in Fig. 2c. These results are in agreement with previous reports^15^,^16^,^18^,^26^. Figure 2e shows the frequency shifts as a function of the lift height. The dashed red line represents the fitted curve using Equation (3). From the fitting, the calculated magnetic moment of the as-measured nanoplatelet was ~1.2 × 10^−16^ *emu*. In addition, we note that some nanoparticles did not show any MFM frequency contrast even through measured at small lift height (Figure S2), suggesting that the nanoparticles composition in these cases may be partially or completely nonmagnetic in nature. These nanoparticles are likely to be the FeOOH phase, confirming the similar morphology but non-magnetic nature of these nanoparticles compared to SrFe_12_O_19_. The results further confirm the frequency shift originated from the magnetic interaction alone.

**Figure 3:**
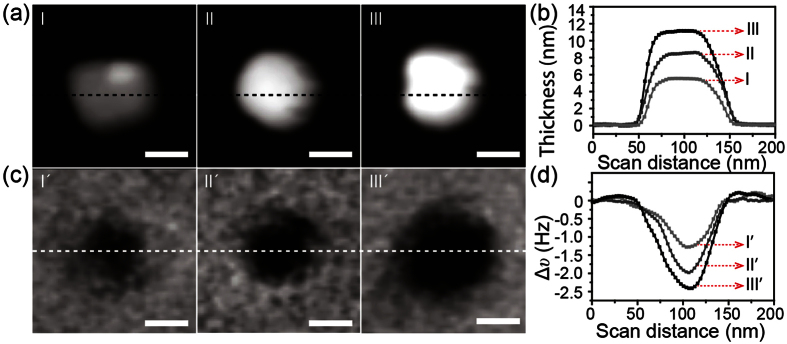
(**a**) AFM height images of three SrFe_12_O_19_ nanoplatelets with different thickness and (**b**) height profiles along the dashed black lines marked in (**a**). (**c**) Corresponding MFM frequency images and (**d**) frequency shift profiles along the dashed white lines marked in (**c**). All scale bars are 50 nm.

As the magnetic moments of the Fe^3+^ ions lie along the c-axis and are coupled by super-exchange interactions through O^2−^ ions (Fig. 1a), MFM frequency imaging was further performed to characterize the magnetic properties of SrFe_12_O_19_ nanoplatelets with different thickness. Figure 3a shows the AFM height images of three SrFe_12_O_19_ nanoplatelets with different thickness. The height images clearly reveal the physical dimensions of the nanoplatelets, and the thickness of the nanoplatelets (5.6 nm, 8.4 nm and 11.2 nm for nanoplatelet I, II, and III, respectively) can be easily obtained from the height profiles (Fig. 3b). The MFM frequency images of these nanoplatelets are shown in Fig. 3c. As can be seen, the contrast in MFM frequency images is enhanced as the thickness of nanoplatelet increases. It is even more evident from Fig. 3d, which shows frequency shift profiles taken along the dashed white line marked in the MFM frequency images in Fig. 3c. The negative frequency shift of the SrFe_12_O_19_ nanoplatelet with a thickness of 8.4 nm increased ~54% (from −1.28 Hz to −1.97 Hz) compared to that of the nanoplatelet with a thickness of 5.6 nm. It further increased by ~89% as the thickness of the nanoplatelet increased to 11.2 nm (from −1.28 Hz to −2.42 Hz). These data suggest that the magnetic moment of SrFe_12_O_19_ nanoplatelets increase as the thickness of the nanoplatelet increases.

**Figure 4:**
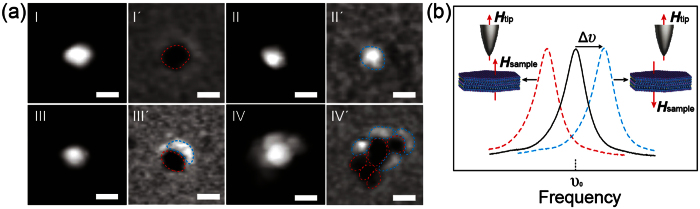
(**a**) AFM height images of three individual SrFe_12_O_19_ nanoplatelets and one SrFe_12_O_19_ nanoplatelet aggregate and their corresponding MFM frequency images. All scale bars are 100 nm. (**b**) Schematic representation of the changes in resonance frequency (solid black curve) of the AFM cantilever due to the magnetic force gradient; in this case, the frequency shift (dashed red curve) reflects an attractive force gradient, and the frequency shift (dashed blue curve) reflects an repulsive force gradient.

Moreover, the MFM frequency imaging of the SrFe_12_O_19_ nanoplatelets shows that although the nanoplatelets had similar lateral size, their MFM frequency contrast can be totally different (Figure S3). Figure 4a represents four typical frequency contrasts. As can be seen, the frequency contrast can be dark (Fig. 4a-I´), bright (Fig. 4a-II´) as well as a combination of dark and bright (Fig. 4a-III´) for individual nanoplatelets and a combination of different dark and bright (Fig. 4a-IV´) for nanoplatelet aggregates.

## Discussion

The contrast in MFM frequency image can be explained using Equation (1), it is clear that the frequency shift shows a negative correlation to the magnetic force gradient. Consequently, a dark contrast (Δυ < 0, dashed red curve in Fig. 4b) in MFM frequency image should be observed when an attractive force is applied to the magnetized probe; on the contrary, a bright contrast (Δυ > 0, blue dashed curve in Fig. 4b) should be observed when a repulsive force is applied to the magnetized probe. In the present study, dark and bright contrasts of the SrFe_12_O_19_ nanoplatelets are observed, indicating that attractive force and repulsive force are detected in the nanoplatelets, respectively. Thus, the appearance of dark and bright contrasts of the nanoplatelet aggregates in the MFM frequency image clearly reveals the magnetization directions of the nanoplatelets in the aggregates. Furthermore, the contrast inversion can be observed by reversing the probe magnetization direction (Figure S4). The fact that the force directions are reversed through switching the probe magnetization directions suggests that the magnetization direction of the nanoplatelets should be constant. These results further confirm that the contrasts in MFM frequency images are came from the ferrimagnetic nature of the SrFe_12_O_19_ nanoplatelets.

**Figure 5:**
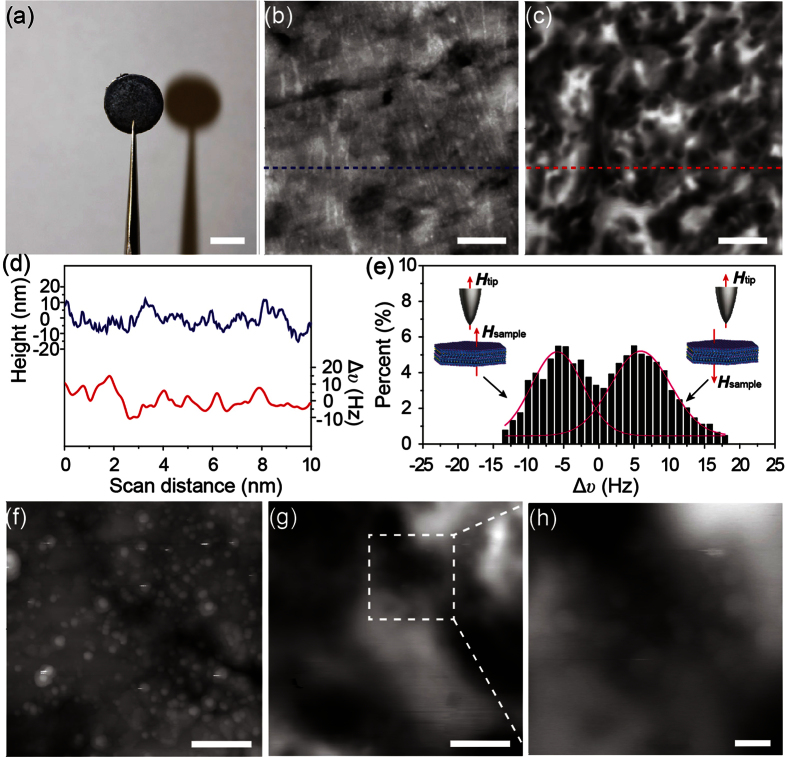
(**a**) Digital camera image of the compacted SrFe_12_O_19_ pellet. Scale bar is 4 mm. (**b**) AFM height image and (**c**) the corresponding MFM frequency image of the surface of the compacted pellet. Scale bars are 2 μm. (**d**) Height and frequency shift profiles measured along the dashed lines marked in height image (**b**) and frequency image (**c**). (**e**) Frequency shift distribution obtained from the MFM frequency image (**c**). (**f**) Zoom-in AFM height image and (**g**) the corresponding MFM frequency image. Scale bars are 500 nm. (**h**) A close-up of the MFM frequency image, corresponding to the dashed white square in (**g**). Scale bar is 100 nm.

After successful characterization of individual SrFe_12_O_19_ nanoplatelets, MFM was then employed to characterize a compacted SrFe_12_O_19_ pellet (Fig. 5a), produced by Spark Plasma Sintering of the as-synthesized hexaferrite nanoplatelets^22^. Figure 5b presents a typical AFM height image of the compacted pellet. The height image clearly reveals the polished surface structure of the compacted pellet. The corresponding MFM frequency image is shown in Fig. 5c. The presence of domains appeared in different contrasts, indicating that different directions of forces are detected at the surface of the compacted pellet. These provide further evidence on local magnetization of the compacted pellet being responsible for the contrast in the MFM frequency image. The observed domains in the MFM frequency image certainly originate from the magnetic domains, as no such structures are observed in the AFM height image (Fig. 5d). The frequency shift distribution of the domains clearly shows two distinct populations of frequency shifts (Fig. 5e), which correspond to the observed dark and bright contrasts in MFM frequency image in Fig. 5c. A zoom-in AFM height image is shown in Fig. 5f. The bright platelet-like structure in the height image indicates that there are individual SrFe_12_O_19_ nanoplatelets on the surface of the compacted pellet. The corresponding MFM frequency image shown in Fig. 5g clearly shows the local magnetization in the compacted pellet. Figure 5h presents a close-up of the MFM frequency image, corresponding to the dashed white square in Fig. 5g, clearly revealing the tiny magnetic structures. These results confirm that MFM can be used to characterize the magnetic properties of complicate magnetic materials.

In conclusion, we demonstrated the applicability of MFM for quantitative imaging of the magnetic nanostructures of SrFe_12_O_19_. By analysis of the MFM frequency shifts, the magnetic moment (~10^−16^ *emu*) of individual SrFe_12_O_19_ nanoplatelets was obtained. In addition, we observed that the magnetic moment of SrFe_12_O_19_ nanoplatelet increases as the thickness of nanoplatelet increases. Furthermore, the magnetization directions of the SrFe_12_O_19_ nanoplatelets in the aggregates can be clearly identified by the contrasts in MFM frequency imaging. Moreover, MFM frequency imaging clearly reveals the magnetic domains of a compacted SrFe_12_O_19_ pellet. Our MFM measurement of the magnetic properties of SrFe_12_O_19_ nanostructures opens up a useful means for the fundamental understanding of the intrinsic magnetic properties of magnetic nanostructures.

## Methods

### Synthesis

The SrFe_12_O_19_ sample was prepared through supercritical synthesis in a flow reactor. Iron nitrates (Fe(NO_3_)_3_·9H_2_O) and strontium nitrates (Sr(NO_3_)_2_) were dissolved in deionized water to obtain a precursor solution with Fe/Sr ratio equal to 1. The precursor solution was then pumped into the supercritical reactor, set at a temperature of 390 °C and a pressure of 250 bar. Then the collected sample was centrifuged, and washed with 2M HNO_3_ solution to remove carbonates present in the as-synthesized sample. Finally, the sample was washed in water and ethanol, and dried in air.

### Compaction

The SrFe_12_O_19_ nanoplatelets were compacted into a dense pellet of 8 mm diameter and around 1 mm thickness. The compaction was performed under vacuum, on a Spark Plasma Sintering system, Syntex Inc. 1500 model, Dr. Sinter Lab^TM^. After compaction, the protective graphite paper surrounding the pellet was removed by polishing, obtaining a smoothed surface SrFe_12_O_19_ pellet.

### Characterization

TEM imaging was conducted using a Phillips CM20 operated at 200 kV. XRD patterns of the as-synthesized samples were collected on a Rigaku SmartLab diffractometer (Rigaku, Japan) using crossbeam optics and a Ge(220) × 2 monochromator to produce Cu K_α1_ radiation. In order to extract crystallographic information, Rietveld refinement was performed on the powder diffraction pattern using the *Fullprof Suite* software. The *M-H* Hysteresis loop was measured at 300 K with a Quantum Design Physical Property Measurement System equipped with a VSM. MFM measurements were performed with a commercial AFM instrument (Dimension Icon, Bruker) under ambient conditions (temperature, 24 °C; relative humidity, 44%). Commercial rectangular silicon cantilever coated with a Co/Cr layer with a resonant frequency υ_0_ of 75 kHz and spring constant *k* of 2.8 N/m (MESP, Bruker) was used for MFM imaging. The magnetic moment m_*t*_ of the magnetic tip is ~10^−13^ *emu*. The tip radius of the magnetic tip is 35 nm. The tip lift height is 10 nm if there is no specific clarification.

## Additional Information

**How to cite this article**: Li, Q. *et al*. Magnetic Properties of Strontium Hexaferrite Nanostructures Measured with Magnetic Force Microscopy. *Sci. Rep.* **6**, 25985; doi: 10.1038/srep25985 (2016).

## Supplementary Material

Supplementary Information
