# Supplementary material for: Magnetic Properties of Strontium Hexaferrite Nanostructures Measured with Magnetic Force Microscopy
Source: Sci Rep. 2016 May 13;6:25985. doi: 10.1038/srep25985 (PMC4865834; doi:10.1038/srep25985)
Supplement: Supplementary Information [file srep25985-s1.pdf]

Supplementary Information

Magnetic Properties of Strontium Hexaferrite Nanostructures Measured with Magnetic Force Microscopy

Qiang Li, Jie Song, Matilde Saura-Múzquiz, Flemming Besenbacher, Mogens Christensen, and Mingdong Dong*

*Correspondence and requests for materials should be addressed to M.D. (dong@inano.au.dk)

Figure S1- S4

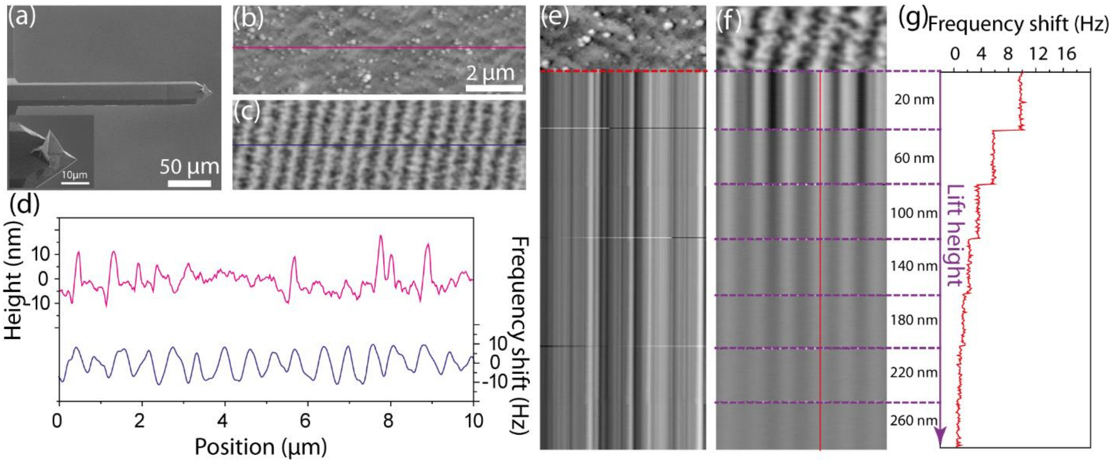

Figure S1. (a) SEM image of the MFM probe used in this study. The SEM image clearly reveals the physical dimensions of the cantilever. The inset image shows the geometry of the tip. (b) AFM height image and (c) the corresponding MFM frequency image (recorded at lift height of 20 nm) of a magnetic recording tape. As can be seen, the MFM frequency image clearly reveals the magnetic domains of the recording tape. (d) Height and frequency shift profiles measured along the solid lines marked in height image (b) and frequency image (c). For insight into the relationship between the MFM frequency shift and lift heights, images were recorded with a disabled slow scan axis of the AFM. In this imaging mode, the AFM tip scans the same position of the sample, as shown in height image (e) and the corresponding frequency image (f). The red dashed line mark in (e) indicates that from that time the slow scan axis of the AFM was disabled. The purple dashed lines marked in (f) indicate the frequency image was recorded at different lift heights with a disabled slow scan axis of the AFM. (g) Frequency shift profile taken along the red solid line marked in (f). As can be seen, the frequency contrast decreases as the lift height increases.

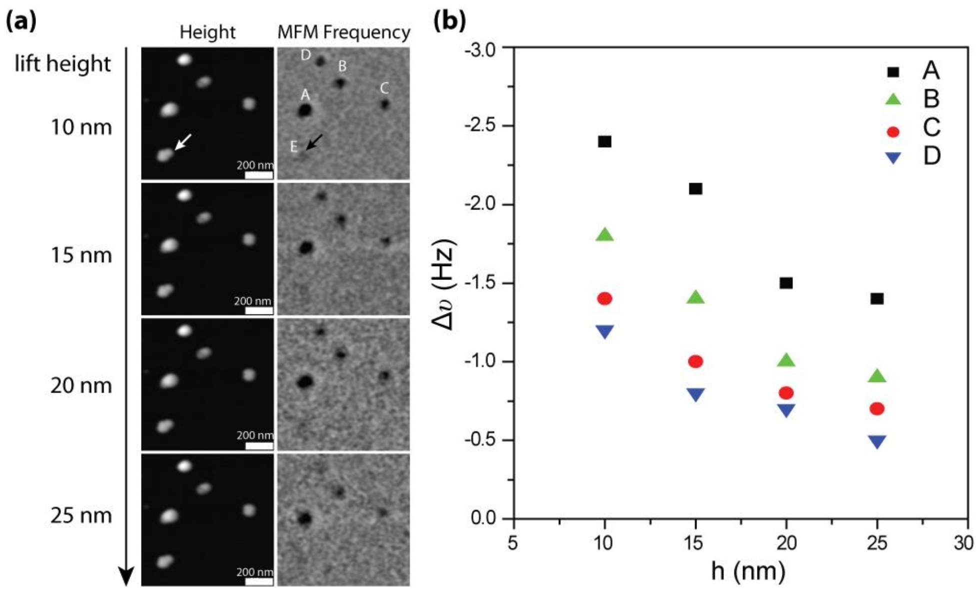

Figure S2. (a) AFM height and corresponding MFM frequency images of five $SrFe_{12}O_{19}$ nanoparticles measured at different lift heights h = 10, 15, 20, 25 nm. It is worth noting that particle E has similar size to the other four particles, but no detectable contrast can be observed from the MFM frequency images even at small lift height, indicating that the composition of particle E may be partially or completely nonmagnetic in nature. This suggests that particle E is not $SrFe_{12}O_{19}$, but FeOOH, explaining its nonmagnetic behavior but similar morphology and size to the $SrFe_{12}O_{19}$ nanoplatelets, as obtained by Rietveld refinement of the XRD data. (b) The plot of frequency shifts as a function of lift height. The solid symbols with different colors correspond to the maximum frequency shifts of the particles obtained from the frequency images in (a).

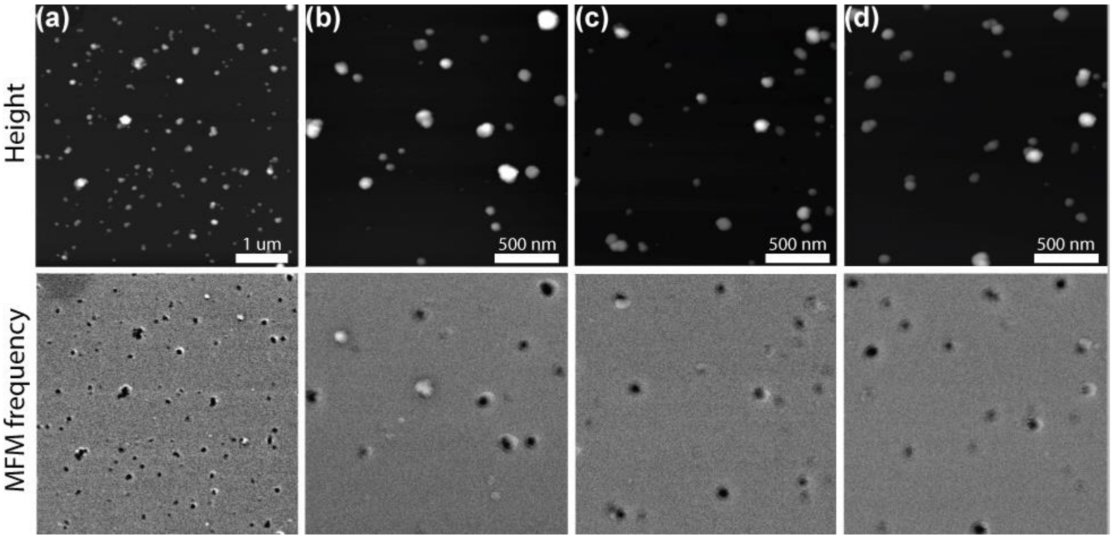

Figure S3. (a) Large scan size of AFM height and corresponding MFM frequency image of $SrFe_{12}O_{19}$ samples. (b-c) Zoom-in AFM height and corresponding MFM frequency images of $SrFe_{12}O_{19}$ samples. MFM frequency shift analysis of the randomly magnetic oriented $SrFe_{12}O_{19}$ samples shows that although the nanoparticles have the similar lateral diameters, there are two distinct populations of frequency shifts.

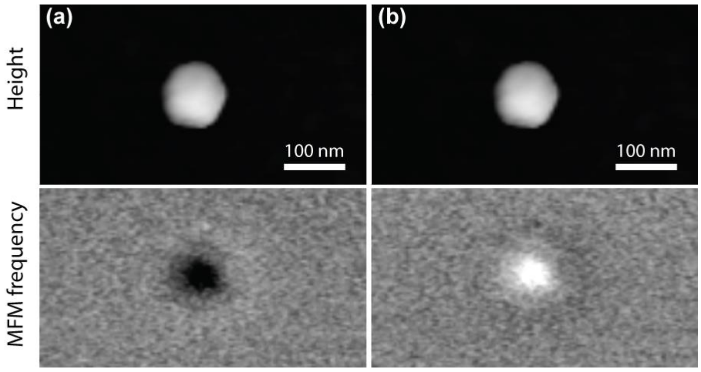

Figure S4. (a) AFM height image and corresponding MFM frequency image of a $SrFe_{12}O_{19}$ nanoplate. (b) AFM height image and corresponding MFM frequency image of the same $SrFe_{12}O_{19}$ nanoplatelet with inverted direction of the magnetization of the AFM tip. As can be seen, the MFM frequency contrast is reversed by changing the tip magnetization direction, indicating that the contrast in the MFM frequency image is originated from the ferrimagnetic nature of the $SrFe_{12}O_{19}$ nanoplate.
